# Association of *Toll*-*like receptor 4 (TLR4)* with chronic plaque type psoriasis and psoriatic arthritis

**DOI:** 10.1007/s00403-016-1620-4

**Published:** 2016-02-01

**Authors:** Rh. Ll. Smith, H. L. Hébert, J. Massey, J. Bowes, H. Marzo-Ortega, P. Ho, N. J. McHugh, J. Worthington, A. Barton, C. E. M. Griffiths, R. B. Warren

**Affiliations:** The Dermatology Centre, Salford Royal NHS Foundation Trust, University of Manchester, Manchester Academic Health Science Centre, Manchester, M6 8HD UK; Arthritis Research UK Centre for Genetics and Genomics, Centre for Musculoskeletal Research, University of Manchester, Manchester Academic Health Science Centre, Manchester, UK; NIHR-Leeds Musculoskeletal Biomedical Research Unit, Leeds Institute of Molecular Medicine, University of Leeds, Leeds, UK; The Kellgren Centre for Rheumatology, Central Manchester Foundation Trust, Manchester, UK; Royal National Hospital for Rheumatic Diseases and Department of Pharmacy and Pharmacology, University of Bath, Bath, UK; NIHR Manchester Musculoskeletal Biomedical Research Unit, Central Manchester Foundation Trust, Manchester Academic Health Science Centre, Manchester, UK

**Keywords:** Psoriasis, Psoriatic arthritis, TLR4, Genetic susceptibility

## Abstract

Family studies have provided overwhelming evidence for an underlying genetic component to psoriasis. Toll-like receptors (TLRs) are key transmembrane proteins in both the innate and adaptive immune responses which are known to be integral processes in psoriasis. Recent functional studies support this notion having suggested a role for *TLR4* in the pathogenesis of psoriasis. Furthermore a missense polymorphism in the *TLR4* gene has been associated with a number of autoimmune conditions, including Crohn diseases, making *TLR4* a viable candidate gene for investigation. The aim of this study was to investigate polymorphisms across the *TLR4* region with a high-density single nucleotide polymorphism (SNP) panel in a large cohort of patients with chronic plaque type psoriasis. Twenty SNPs were successfully genotyped using Sequenom iPLEX Gold platform in 2826 UK chronic plaque type psoriasis patients including subgroup data on presence of confirmed psoriatic arthritis (*n* = 1839) and early-onset psoriasis (*n* = 1466) was available. Allele frequencies for psoriasis patients were compared against imputed Wellcome Trust Case Control Consortium controls (*n* = 4861). Significant association was observed between a missense variant rs4986790 of *TLR4* (Asp229Gly) and plaque type psoriasis (*p* = 2 × 10^−4^) which was also notable in those with psoriatic arthritis (*p* = 2 × 10^−4^) and early-onset psoriasis (*p* = 8 × 10^−4^). We present data suggestive of an association between a functional variant and an intronic variant of *TLR4* and chronic plaque type psoriasis and psoriatic arthritis. However, validation of this association in independent cohorts will be necessary.

## Introduction

Family studies have provided incontrovertible evidence in support of an underlying genetic component to the aetiology of psoriasis. Recent meta-analyses of genome-wide association studies [14–16] and other high-density single nucleotide polymorphism (SNP) panel data [17] have confirmed 41 genetic loci in psoriasis yet it is widely accepted that a considerable portion of the disease’s heritability remains unidentified. Research focusing on the immunological processes underlying psoriasis has also provided an invaluable insight into its pathogenesis. Toll-like receptors (TLRs) are transmembrane proteins expressed on immune cells that recognise conserved regions of both endogenous and exogenous molecules as part of the innate and adaptive immune responses. Crucially, TLRs are expressed in the skin and are differentially expressed in involved psoriasis skin when compared to uninvolved skin [2, 4]. Additionally, recent studies of imiquimod psoriasis mouse models have shown TLR-dependent pathways to be key in both the formation [7] and maintenance [19] of psoriasis plaques.

Toll-like receptor 4 is speculated to be a trigger of apoptosis and interacts with *TLR2* in the autoimmune pathway. Furthermore, a recent study has demonstrated an increase in *TLR4* expression on peripheral blood mononuclear cells of psoriasis patients compared to controls [6]. Polymorphisms within *TLR4* have been associated with a number of autoimmune conditions, genetically linked to psoriasis, including Crohn disease [13], vitiligo [10] and ulcerative colitis [13] as well as a wide range of other diseases including Behcet’s involving arthritis [8], atherosclerosis [5] and endotoxin hyporesponsiveness [1]. Therefore, due to the increasing evidence suggestive of a role for *TLR4* in the pathogenesis of psoriasis, we investigated polymorphisms across the gene with a high-density SNP panel in a cohort of 2826 UK patients with chronic plaque type psoriasis.

## Materials and methods

Of these patients, 1839 had confirmed psoriatic arthritis with the status in the remainder unknown. Age of onset data was available for 2137 patients with 1466 defined as early-onset (age of onset <40 years, mean 20.8 ± SD 9.8 years, range 0–39 years); 671 as late-onset (age of onset ≥40 years, mean 50.6 ± SD 9.1 years, range 40–81 years) and 689 where the age of onset was unknown. The early-onset cohort consisted of 861 PsA cases (58.7 %), whilst the late-onset cohort consisted of 423 PsA cases (63.0 %).

A panel of 22 SNPs were selected across the *TLR4* region (inclusive of 10 kbp up and downstream) including tagging SNPs (*r*^2^ > 0.9), previously associated markers and proxy markers in case of SNP failure using the Tagger application within Haploview [3]. This panel of SNPs captured 97 % of alleles with MAF > 1 % (HapMap Phase III Caucasian population, Build 36 database, May 2010).

In total, 2983 UK chronic plaque type psoriasis patients were genotyped using a Sequenom^®^ MassArray™ platform and iPLEX Gold chemistry. Quality control (QC) of the genotyping included removal of samples and markers with a success rate <80 %, a minor allele frequency <1 %, or a Hardy–Weinberg equilibrium *p* value <0.0001. Control information on these loci was obtained from imputation of WTCCC GWAS control data incorporating 2889 samples from the 1958 British Birth Cohort (1958BC) and 2834 samples from the UK Blood Services collection (NBS) [18] using IMPUTE2 algorithm [9]. Statistical analysis of the data was performed using the PLINK (v1.07) statistical software package [12], using an allelic association test.

## Results

Twenty SNPs (Table 1) were successfully genotyped (two assays failed due to poor cluster plots: rs10983755 and rs1927911) in 2826 UK chronic plaque type psoriasis patients (success rate = 94.7 %) including those with confirmed psoriatic arthritis (*n* = 1839) and those known to be early-onset (*n* = 1466) although these groups were not mutually exclusive. Following imputation of the WTCCC control cohort and QC, allele frequencies were compared against a total of 4861 imputed controls with information scores greater than 0.97 at each loci.

**Table 1:** Association of polymorphisms within the toll-like receptor 4 gene region with clinical subtypes of psoriasis

SNP	Chr 9bp position	A1/A2	Function	Imputed controls (*n* = 4861)		Patients with psoriasis (*n* = 2826)		
				MAF	Imp.Info. score	MAF	Allelic *p* (SE)	OR (95 % CI)
rs10818070	119496316	A/G	Intergenic	0.07	1.000	0.08	0.0419 (0.06)	1.13 (1.01–1.28)
rs10759930	119501442	T/C	Upstream	0.38	1.000	0.36	0.0360 (0.03)	0.93 (0.87–1.00)
rs2737191	119502536	G/A	Upstream	0.29	1.000	0.29	0.6362 (0.04)	1.02 (0.95–1.09)
rs10116253	119504141	C/T	Upstream	0.25	0.999	0.26	0.7027 (0.04)	1.02 (0.94–1.09)
rs10759932	119504965	C/T	Upstream	0.13	0.999	0.14	0.1231 (0.05)	1.08 (0.98–1.19)
*rs12344353*	*119508470*	*C/T*	*Intronic*	*0.06*	*0.998*	*0.07*	*0.0005 (0.07)*	*1.27 (1.11–1.45)*
rs11536871	119510319	C/A	5′ UTR/Intronic	0.04	1.000	0.04	0.1132 (0.08)	1.14 (0.97–1.34)
rs11536878	119511374	A/C	Intronic	0.12	1.000	0.12	0.3100 (0.05)	0.95 (0.86–1.05)
rs1927907	119512585	T/C	Intronic	0.13	0.997	0.14	0.1468 (0.05)	1.07 (0.98–1.18)
rs2149356	119514020	T/G	Intronic	0.31	0.997	0.33	0.0118 (0.04)	1.10 (1.02–1.17)
*rs4986790*	*119515123*	*G/A*	*Missense*	*0.05*	*1.000*	*0.07*	*0.0002 (0.07)*	*1.30 (1.13–1.48)*
rs7873784	119518757	C/G	3′ UTR	0.16	0.998	0.15	0.5298 (0.05)	0.97 (0.89–1.06)
rs1927906	119519936	C/T	Downstream	0.09	1.000	0.11	0.0053 (0.06)	1.17 (1.05–1.30)
rs1554973	119520633	C/T	Downstream	0.25	1.000	0.26	0.1249 (0.04)	1.06 (0.98–1.14)
rs7044464	119521218	A/T	Downstream	0.16	0.999	0.15	0.6902 (0.05)	0.98 (0.90–1.08)
rs7037225	119523460	T/C	Downstream	0.15	1.000	0.16	0.0958 (0.05)	1.08 (0.99–1.18)
rs913930	119523830	G/A	Downstream	0.37	1.000	0.37	0.7308 (0.03)	1.01 (0.95–1.08)
rs2183016	119525036	C/A	Intergenic	0.16	1.000	0.15	0.5178 (0.05)	0.97 (0.89–1.06)
rs1927905	119525129	C/T	Intergenic	0.06	1.000	0.05	0.2272 (0.07)	0.91 (0.79–1.06)
rs10759934	119528817	T/A	Downstream	0.48	0.972	0.47	0.0622 (0.03)	0.94 (0.88–1.00)

SNP	PSA confirmed patients (*n* = 1839)			Early-onset psoriasis (*n* = 1466)			Late-onset psoriasis (*n* = 671)		
	MAF	Allelic *p* (SE)	OR (95 % CI)	MAF	Allelic *p* (SE)	OR (95 % CI)	MAF	Allelic *p* (SE)	OR (95 % CI)
rs10818070	0.09	0.0272 (0.07)	1.17 (1.02–1.34)	0.09	0.0269 (0.08)	1.18 (1.02–1.37)	0.08	0.5374 (0.11)	1.07 (0.87–1.32)
rs10759930	0.36	0.0148 (0.04)	0.91 (0.84–0.98)	0.37	0.3513 (0.04)	0.96 (0.88–1.05)	0.36	0.2098 (0.06)	0.93 (0.82–1.04)
rs2737191	0.29	0.7939 (0.04)	1.01 (0.93–1.10)	0.29	0.9495 (0.05)	1.00 (0.91–1.09)	0.30	0.5233 (0.06)	1.04 (0.92–1.18)
rs10116253	0.26	0.2268 (0.04)	1.06 (0.97–1.15)	0.25	0.6751 (0.05)	0.98 (0.89–1.08)	0.25	0.9297 (0.07)	0.99 (0.87–1.13)
rs10759932	0.14	0.0548 (0.06)	1.11 (1.00–1.24)	0.13	0.4063 (0.06)	1.05 (0.93–1.19)	0.14	0.3620 (0.08)	1.08 (0.91–1.28)
*rs12344353*	*0.07*	*0.0007 (0.08)*	*1.30 (1.12–1.51)*	*0.07*	*0.0009 (0.08)*	*1.32 (1.12–1.56)*	*0.07*	*0.0754 (0.12)*	*1.23 (0.98–1.55)*
rs11536871	0.04	0.1840 (0.10)	1.14 (0.94–1.37)	0.05	0.0755 (0.10)	1.20 (0.98–1.47)	0.04	0.9280 (0.15)	1.01 (0.76–1.36)
rs11536878	0.12	0.8061 (0.06)	0.99 (0.88–1.11)	0.11	0.1440 (0.07)	0.91 (0.80–1.03)	0.12	0.4645 (0.09)	0.94 (0.78–1.12)
rs1927907	0.14	0.0765 (0.06)	1.11 (0.99–1.23)	0.14	0.4211 (0.06)	1.05 (0.93–1.19)	0.14	0.4621 (0.09)	1.07 (0.90–1.26)
rs2149356	0.34	0.0042 (0.04)	1.13 (1.04–1.22)	0.33	0.1048 (0.05)	1.08 (0.98–1.18)	0.33	0.1741 (0.06)	1.09 (0.96–1.23)
*rs4986790*	*0.07*	*0.0002 (0.08)*	*1.33 (1.14–1.55)*	*0.07*	*0.0008 (0.08)*	*1.33 (1.13–1.57)*	*0.07*	*0.0181 (0.12)*	*1.31 (1.05–1.65)*
rs7873784	0.16	0.7570 (0.05)	1.02 (0.92–1.13)	0.14	0.0531 (0.06)	0.89 (0.79–1.00)	0.16	0.4379 (0.08)	1.06 (0.91–1.24)
rs1927906	0.11	0.0047 (0.06)	1.20 (1.06–1.36)	0.11	0.0156 (0.07)	1.18 (1.03–1.36)	0.11	0.0630 (0.09)	1.19 (0.99–1.44)
rs1554973	0.27	0.0251 (0.04)	1.10 (1.01–1.20)	0.25	0.7596 (0.05)	1.02 (0.92–1.12)	0.27	0.0660 (0.07)	1.13 (0.99–1.28)
rs7044464	0.16	0.7371 (0.05)	1.02 (0.92–1.13)	0.14	0.1082 (0.06)	0.91 (0.81–1.02)	0.16	0.4379 (0.08)	1.06 (0.91–1.24)
rs7037225	0.17	0.0814 (0.05)	1.10 (0.99–1.22)	0.16	0.2350 (0.06)	1.07 (0.96–1.20)	0.17	0.2129 (0.08)	1.10 (0.95–1.29)
rs913930	0.37	0.9862 (0.04)	1.00 (0.92–1.08)	0.37	0.8602 (0.04)	1.01 (0.92–1.10)	0.36	0.8490 (0.06)	0.99 (0.88–1.11)
rs2183016	0.16	0.7878 (0.05)	1.01 (0.91–1.13)	0.14	0.0577 (0.06)	0.89 (0.79–1.00)	0.16	0.4963 (0.08)	1.06 (0.90–1.23)
rs1927905	0.05	0.2980 (0.09)	0.91 (0.77–1.08)	0.05	0.1637 (0.10)	0.87 (0.72–1.06)	0.05	0.8037 (0.13)	0.97 (0.75–1.25)
rs10759934	0.47	0.2435 (0.04)	0.96 (0.88–1.03)	0.47	0.1281 (0.04)	0.94 (0.86–1.02)	0.48	0.6212 (0.06)	0.97 (0.87–1.09)

*PS* psoriasis patients, *PSA* psoriatic arthritis, *Chr 9* chromosome 9, *Bp* base pairs, *SE* standard error, *Imp.Info. Score* imputation information score, *MAF* minor allele frequency, *95* *% CI* 95 % confidence interval, *OR* odds ratio

Significant association was observed in patients with psoriasis at 2 of the 20 SNPs analysed when implementing a Bonferroni adjusted significance threshold of *p* < 0.0025 (rs12344353 *p* = 5 × 10^−4^; rs4986790 *p* = 2 × 10^−4^; italic rows Table 1). However, when accounting for linkage disequilibrium between the markers (independent signals between SNPs defined as *r*^2^ < 0.4) and retaining the most significant SNP, only rs4986790 remained significant (*r*^2^ of 0.96 with rs12344353) with risk conferred by carriage of the minor allele (*p* = 2 × 10^−4^; OR = 1.30, 95 % CI 1.13–1.48, Table 1). This was confirmed when conditioning a logistic regression analysis of all other markers on rs4986790 revealing there to be no significant associations at the defined threshold (data not shown).

Notably, rs4986790 is a missense variant located in exon 3 of *TLR4* (chromosome 9) and encodes an aspartic acid to glycine substitution (Asp229Gly). This particular polymorphism has been shown to interrupt TLR4-mediated lipopolysaccharide signalling in mice and further alters lipopolysaccharide responsiveness in humans—thereby altering their ability to respond to environmental stressors [1]. In light of this, it is interesting to consider whether this variant may have a role in different manifestations of the disease, such as psoriatic arthritis and early onset compared to late-onset, as psoriasis is known to have a wide range of environmental triggers, and the increased minor allele frequency within the patients—which is postulated to drive this altered response to lipopolysaccharide—would support such a hypothesis.

Variant rs4986790 showed strongest evidence of association within those patients with confirmed psoriatic arthritis (*n* = 1839, *p* = 2 × 10^−4^, Table 1) whereas no association was observed when comparing those without definitive information on presence of psoriatic arthritis against controls (*n* = 987 patients, *p* = 0.0462, OR = 1.22, 95 % CI 1.00–1.49). However, when the dataset was dichotomised according to age of onset, the early-onset cohort was significantly associated with rs4986790 (*p* = 8 × 10^−4^, OR = 1.33; 95 % CI 1.13–1.57, Table 1), but the late-onset cohort was not significant at the adjusted threshold (*p* = 0.0181, OR = 1.31 95 % CI 1.05–1.65). Given that the proportion of confirmed PsA cases in each dichotomised cohort was roughly the same, these results appear to suggest that PsA is not driving this association. However, the lack of association in late-onset psoriasis could be due to lack of power caused the low sample size of the cohort overall (*n* = 671) compared to early-onset psoriasis (*n* = 1466), as well as the lower number of confirmed PsA samples (*n* = 423 vs 861).

## Discussion

In support of the data, a recent investigation of functional polymorphisms within *TLR2* and *4* reported an increased frequency of rs4986790 SNP in Turkish vitiligo patients (*n* = 100) when compared to healthy controls (*n* = 100) with a similar minor allele frequency in the control cohorts of both studies [10]. Furthermore, there is speculation that these diseases are genetically linked with a recent meta-analysis reporting the diseases to harbour common susceptibility loci in the Chinese Han population—predominantly within the HLA region on chromosome 6 [20]. A meta-analysis of other autoimmune conditions linked to psoriasis including Crohn disease and ulcerative colitis also found the minor allele of rs4986790 to confer increased disease risk [13].

It is worth noting that to date, large genome-wide association and high-density SNP panel studies which include rs4986790 are yet to report any significant association with psoriasis or psoriatic arthritis. This could be due to issues in the past with heterogeneous cohorts, as different manifestations of psoriasis such as chronic plaque and generalised pustular type are known to have different genetic signatures [11]. Another explanation is that past failures to find an association could just be an artefact of random sampling. Furthermore, a recent study mapping *cis*-acting expression quantitative trait loci (eQTLs) in psoriasis using normal skin from 57 healthy controls, and both involved and uninvolved skin from 53 psoriasis patients did not report any significant associated SNPs tagging *TLR4* eQTLs in their analysis (*p* < 9 × 10^−7^) [17]. Additionally, we have previously reported an association between *TLR4* loci (rs10759932; rs7044464; rs752998) and early-onset psoriasis (age of onset <40 years) in 664 patients and 566 healthy population based controls. We were able to replicate the associations in this study although the associated rs4986790 SNP was different to that tested in the previous study (unpublished data).

Therefore the associations reported in our data should be treated with caution. Nonetheless, it is of significant interest that a functional missense variant in *TLR4*, known to be associated with conditions genetically linked to psoriasis, shows significant association with psoriasis and psoriatic arthritis in this study. Validation of these associations would be crucial in confirming any association of *TLR4* with psoriasis and/or psoriatic arthritis with particular focus given to accurate phenotyping; including of age of onset and associated presence or absence (confirmed by a rheumatologist) of arthritis [2, 4, 6].
